# A population-based analysis of increasing rates of suicide mortality in Japan and South Korea, 1985–2010

**DOI:** 10.1186/s12889-016-3020-2

**Published:** 2016-04-23

**Authors:** Sun Y. Jeon, Eric N. Reither, Ryan K. Masters

**Affiliations:** Department of Sociology and Yun Kim Population Research Laboratory, Utah State University, 0730 Old Main Hill, 84322 Logan, UT USA; Department of Sociology, University of Colorado Boulder, UCB 327 Ketchum Hall 264, 80309 Boulder, CO USA

**Keywords:** Age-period-cohort analysis, Intrinsic estimator model, Japan, South Korea, Suicide

## Abstract

**Background:**

In the past two decades, rates of suicide mortality have declined among most OECD member states. Two notable exceptions are Japan and South Korea, where suicide mortality has increased by 20 % and 280 %, respectively.

**Methods:**

Population and suicide mortality data were collected through national statistics organizations in Japan and South Korea for the period 1985 to 2010. Age, period of observation, and birth cohort membership were divided into five-year increments. We fitted a series of intrinsic estimator age-period-cohort models to estimate the effects of age-related processes, secular changes, and birth cohort dynamics on the rising rates of suicide mortality in the two neighboring countries.

**Results:**

In Japan, elevated suicide rates are primarily driven by period effects, initiated during the Asian financial crisis of the late 1990s. In South Korea, multiple factors appear to be responsible for the stark increase in suicide mortality, including recent secular changes, elevated suicide risks at older ages in the context of an aging society, and strong cohort effects for those born between the Great Depression and the aftermath of the Korean War.

**Conclusion:**

In spite of cultural, demographic and geographic similarities in Japan and South Korea, the underlying causes of increased suicide mortality differ across these societies—suggesting that public health responses should be tailored to fit each country’s unique situation.

## Background

**Fig. 1:**
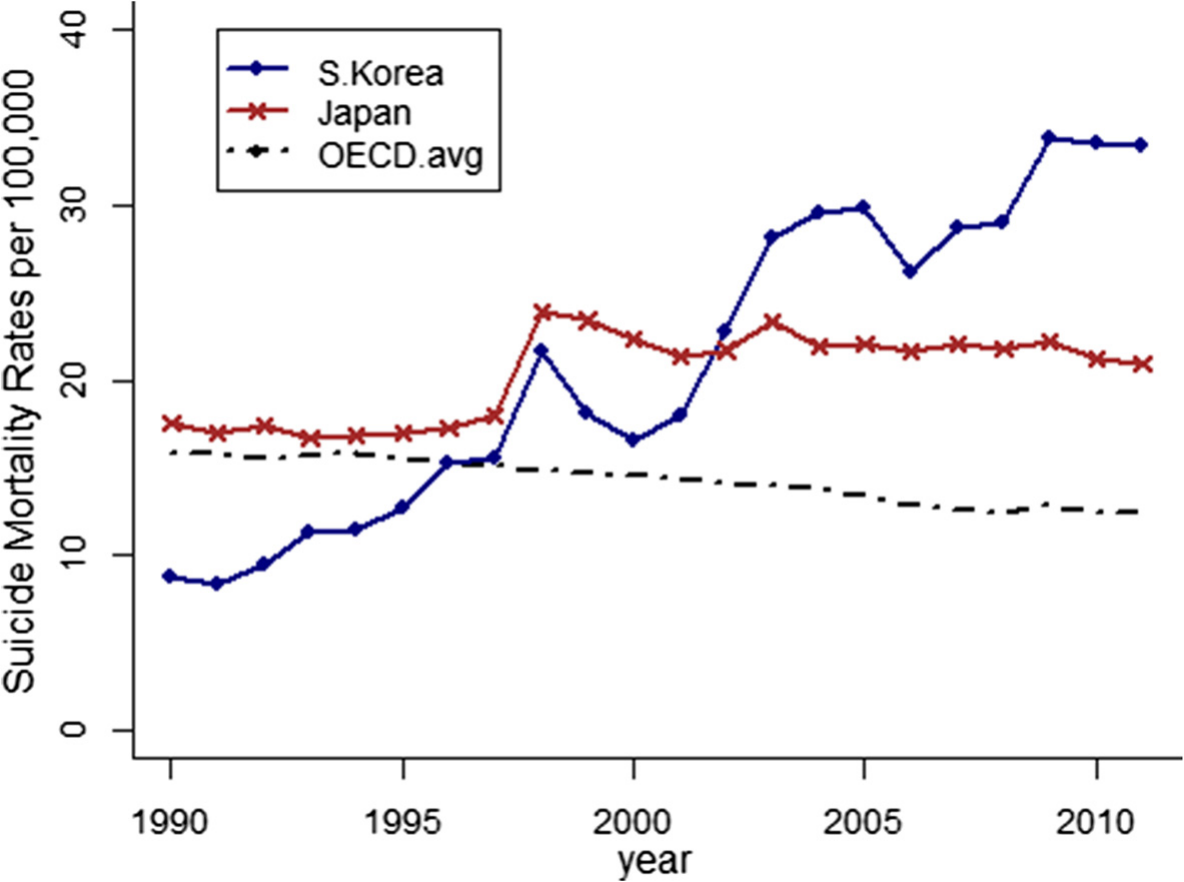
Age-standardized suicide mortality rates in Japan, South Korea and all OECD nations (averaged), 1990–2012 (Source: OECD Factbook. Rates are age-standardized to the 2012 OECD population to account for differences in age structures across countries and over time [1].)

In recent decades, rates of suicide mortality have steadily declined among most member states of the Organization for Economic Cooperation and Development [1]. Unfortunately, Japan and South Korea are notable exceptions to this overall trend. Rates of suicide mortality in Japan spiked in 1998 and remained high thereafter (Fig. 1). In South Korea, the rate of suicide mortality has climbed since 1985, reaching over 30 per 100,000 person-years lived (PYL) in 2010. As a result of these trends, South Korea and Japan now exhibit the two highest rates of suicide mortality among all OECD countries.

Prominent social scientists have argued that elevated suicide rates stem from disturbances to the social equilibrium, such as economic recession or rapid industrial expansion [2–5]. A small group of studies has evaluated the impact of specific social changes on increasing suicide rates in Japan and South Korea. These studies have detected associations between suicide and divorce rates [6], changing patterns of marriage [7] and the Asian economic crisis that impacted both Japan and South Korea in the late 1990s [8].

Another body of scholarship has attempted to isolate the effects of sweeping social changes (i.e., secular trends or period effects) on suicide mortality from factors related to population aging and birth cohort membership. Understanding the relative impact of age, period, and cohort effects is important because it can illuminate the mechanisms responsible for changes in a population’s suicide rates. For instance, Odagiri et al. [9] suggest that strong age effects among middle-aged men in Japan are primarily responsible for increasing suicide rates—not secular changes such as the economic recession. In a similar analysis, Lee and Kim [10] argue that birth cohort membership has made the largest contribution to increasing suicide rates in South Korea. Results from these studies suggest that the underlying causes of increasing suicide mortality may differ in Japan and South Korea, despite geographic, cultural and demographic similarities shared by the two countries.

To better understand increasing rates of suicide mortality in Japan and South Korea, this study extends previous research in three ways: First, whereas some prior research has focused on the effect of one temporal factor such as age [6] or secular changes [8] on suicide in these countries, we analyzed the distinct effects of all three time-related demographic factors (age, period, and birth cohort) on suicide in Japan and South Korea by applying the intrinsic estimator (IE)— an innovative method with desirable statistical properties for age-period-cohort (APC) modeling [11, 12]— to suicide mortality rates. Second, we extend the period of analysis to 2010; this is important because previous research on suicide in either Japan [9] or South Korea [10] using APC modeling is generally limited to the early 2000s, prior to South Korea’s extraordinarily rapid increase in suicide mortality that led to its sharp divergence from other OECD nations, including Japan. Third, by comparing and contrasting the demographic factors that have shaped suicide mortality patterns over the last quarter century in Japan and South Korea, our study reveals commonalities and differences in these neighboring Asian countries, with attendant public health implications for each nation.

## Methods

### Data and measures

Analyzing rates of suicide mortality in South Korea and Japan between 1985 and 2010 required age- and sex-specific data on the number of suicide deaths (numerators) and population counts (denominators) over this period of observation. Cause-specific mortality data for each country’s population (including the number of suicide deaths) was provided by Statistics Korea [13] and the Vital Statistics Survey [14], respectively. Population estimates were provided by national statistics organizations—Statistics Korea and, in Japan, the Ministry of Internal Affairs and Communications.

After securing these data, we divided age from 10 to 80 into fourteen 5-year age groups. For example, the age group 10–14 refers to people aged 10.0 to 14. $\overline{9}$. The youngest age groups (0–4 and 5–9) were excluded because suicide at these ages is extremely rare. We included a total of six periods of observation in our study, which were evenly spaced in 5-year intervals over the period 1985 to 2010. By subtracting age from the period of observation, we derived individuals’ birth cohort membership, which we also grouped into 5-year intervals. We excluded people over the age of 80 from our analysis because both census data and cause-specific mortality data in Japan and South Korea provide an open-ended interval for the eldest age group, making it impossible to derive 5-year birth cohorts. Consequently, we evaluated suicide mortality data for nineteen separate cohorts born between 1905 and 2000 across the time period 1985 to 2010.

### Statistical analysis

#### Classic age-period-cohort models

Age-Period-Cohort (APC) models have been developed to estimate effects for all three temporal factors in studies of cause-specific mortality rates. In the generalized linear APC model, suicide mortality can be written in log-linear regression form [15]:

1 $$ \log \left({E}_{ijk}\right)= \log \left({P}_{ijk}\right)+\mu +{\alpha}_{\mathrm{i}}+{\upbeta}_{\mathrm{j}}+{\upgamma}_{\mathrm{k}} $$

where *E*_*ijk*_ denotes the expected number of suicides which is assumed to follow the Poisson distribution, for the *i*th age group at the *j*th period of observation and the *k*th birth cohort for *i* = *1*,…,*a*, *j* = *1*,…,*p* and *k* = *1*,…,(*a* + *p*-*1*); *P*_*ijk*_ denotes the size of the population for each age-period-cohort combination, which is referred to as the offset term in the log-linear regression; μ is the intercept; α_*i*_ is the age effect in the *i*th age group; β_*j*_ is the period effect in the *j*th observation period; γ_*k*_ is the cohort effect for the *k*th birth cohort, where *k* = *a*-*i* + *j* [12, 16, 17].

Equation (1) is under-identified in traditional ordinary least square (OLS) regression analysis of tabular data because the three temporal factors are linearly related to each other (age = period-cohort), leading to the design matrix X of one less than full rank.^1^ Consequently, a unique solution does not exist and the model has an infinite set of A, P, and C estimates. This under-identification issue is a limitation inherent to classic APC accounting models [18].

Traditional constrained generalized linear models (CGLIM) address APC model under-identification by imposing an equality constraint, usually on two adjacent age, period or cohort estimates [19]. A limitation of CGLIM analysis is that model estimates are often sensitive to the choice of equality constraint [16, 20], leading researchers to rely on strong *a priori* assumptions based on theoretical considerations or other external information [18]. However, unless these assumptions are completely sound, there is substantial risk for model misspecification [16, 19, 21, 22].

With these challenges in mind, we adopted the *intrinsic estimator* (IE) method for APC modeling [12], which identifies a constraint not dependent on *a priori* theoretical assumptions of age, period, or cohort effects, but rather the number of age (*a*) and period (*p*) categories.^2^ We fit IE models to sex- and country-specific suicide mortality data using the *apc*_*ie* module in Stata 13 [23].^3^

## Results

### Descriptive analysis

**Fig. 2:**
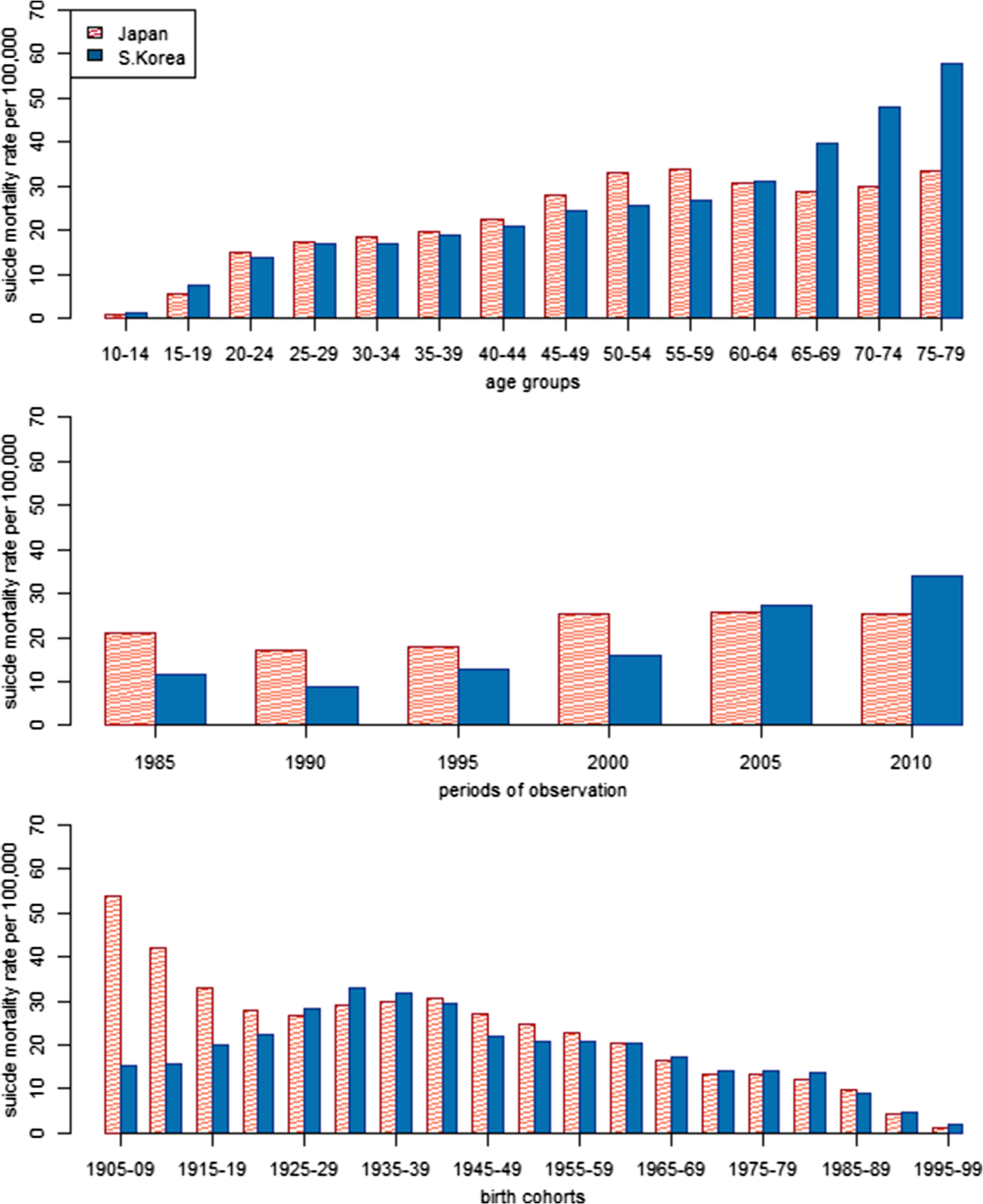
Suicide mortality rates by age, period and birth cohort in Japan and South Korea, 1985–2010

Figure 2 displays suicide mortality rates per 100,000 person-years lived (PYL) for different age groups, periods of observation, and birth cohorts. We calculated these rates by dividing the number of suicide deaths by the population for each age group, period of observation, and birth cohort, and then multiplied by a constant of 100,000 to ease interpretation. In both countries, suicide rates are very low at young ages, but by age 30–34 increase to 18.7 per 100,000 PYL in

**Fig. 3:**
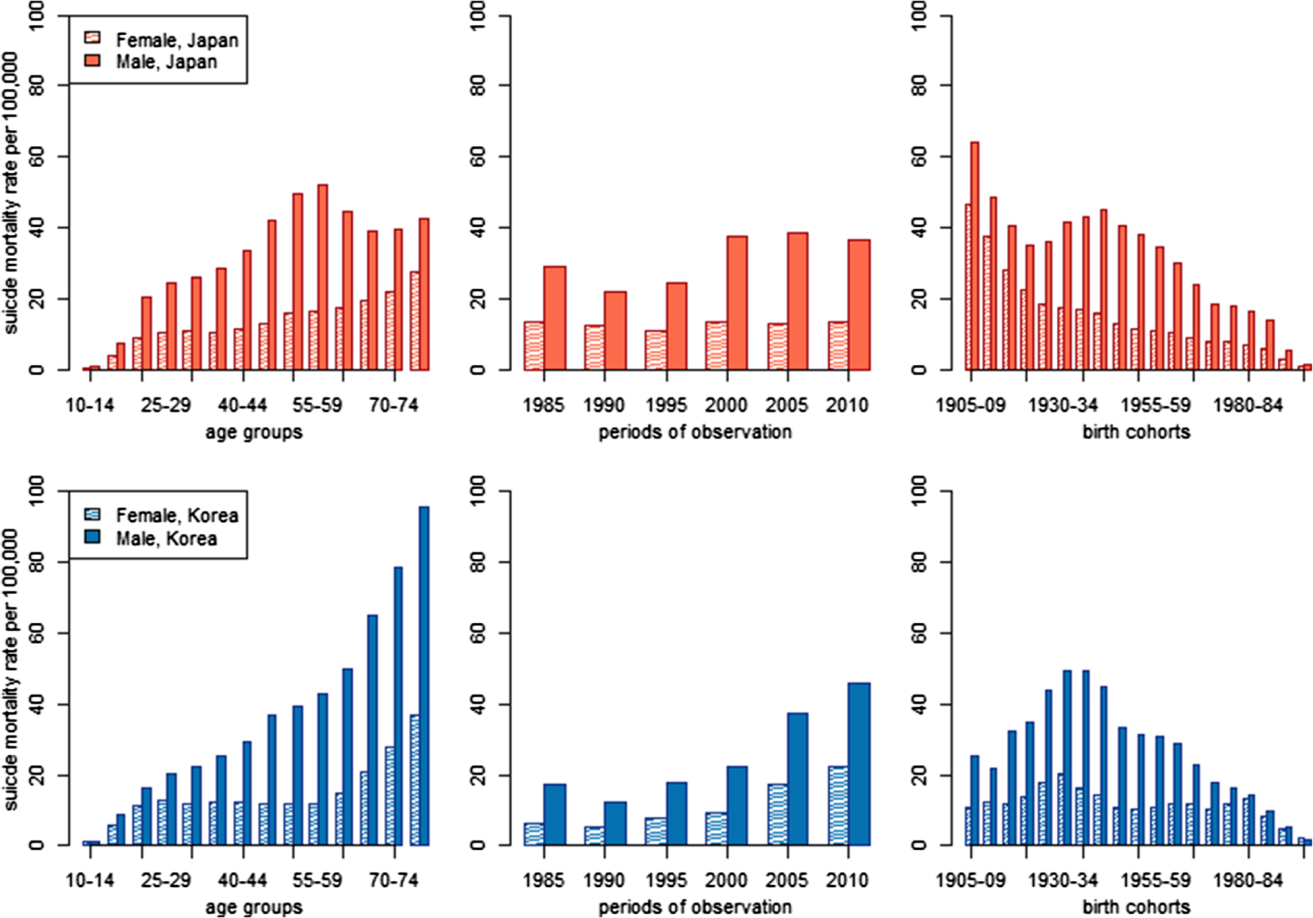
Sex-stratified suicide mortality rates by age, period and birth cohort in Japan and South Korea, 1985–2010

Japan and 17.0 per 100,000 PYL in South Korea. Suicide rates continue to increase through midlife in both countries, but the pace of increase is especially rapid in Japan, where rates peak at age 55–59. Conversely, in South Korea suicide rates increase steadily with age across the entire life course. When disaggregated by gender, it becomes clear that age-specific rates of suicide are far higher among men than women in both countries (Fig. 3). In addition, among women in both countries, suicide rates are low and fairly stable from the 20s through midlife, but subsequently increase during later phases of the life course. This indicates that the midlife peak in suicide mortality in Japan’s general population is driven by distinct suicide patterns among Japanese men.

In the initial period of observation (1985), suicide mortality rates were about two times higher in Japan than South Korea (Fig. 2). Suicide then declined in 1990 in both countries, before rising again later that decade. In Japan, suicide rates spiked between 1995 and 2000, then stabilized at about 26 per 100,000 PYL (26.1 in 2000, 26.2 in 2005 and 25.5 in 2010). Conversely, suicide increased sharply in South Korea after 2000, reaching 35.9 per 100,000 PYL in 2010. Suicide mortality was higher among men than women in each period (Fig. 3). In Japan, suicide rates were fairly stable among women during the entire period of observation, revealing that the spike in suicide in the late 1990s was caused almost entirely by men. Despite very different levels of suicide mortality, men and women in South Korea exhibited similar trends between 1985 and 2010.

Cohorts born between 1925–29 and 1995–99 tended to show remarkably similar levels of suicide mortality in Japan and South Korea (Fig. 2). However, members of older Japanese birth cohorts exhibited much higher suicide rates than their South Korean counterparts. In the oldest birth cohort in our study (1905–09), suicide rates were well over 50 per 100,000 PYL in Japan, but only about 15 per 100,000 PYL in South Korea. Men and women in South Korea showed generally similar patterns of suicide mortality across birth cohorts (Fig. 3). In Japan, cohort-based trends differ by sex; whereas women showed a steady decline in suicide from the oldest to the youngest birth cohorts, men exhibited a decline for cohorts born between 1905 and the mid-1920s, followed by an increase for cohorts born through the mid-1940s, and then another decline. Trends among birth cohorts reflect those observed among different age groups, demonstrating the need to disentangle age, period and cohort effects through multivariate modeling, which we turn to next,

### APC estimates from IE models

**Fig. 4:**
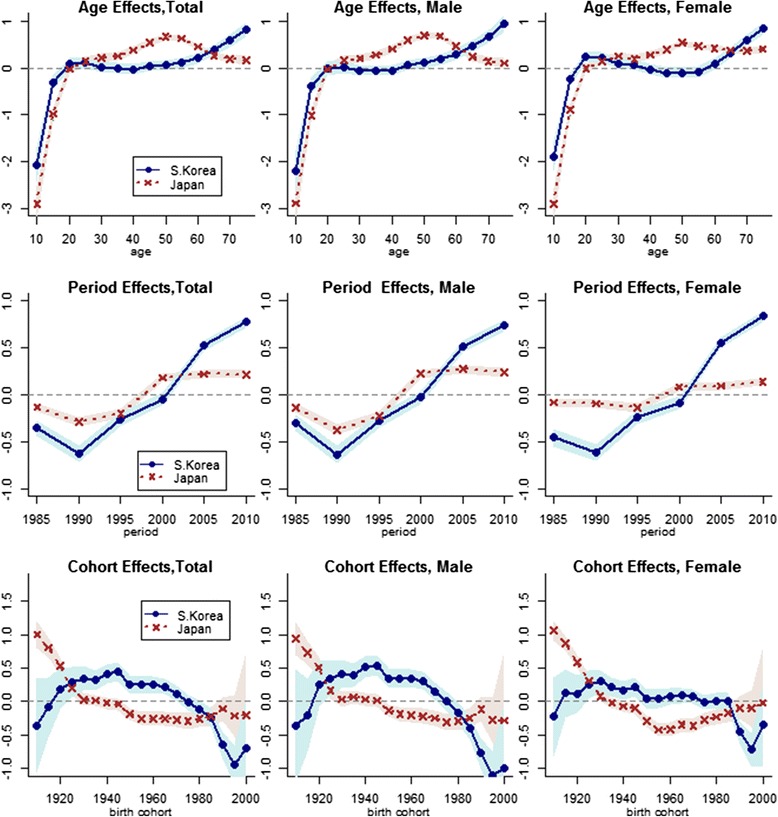
Estimates from intrinsic estimator models of age, period, and cohort effects on suicide mortality in Japan and South Korea, 1985–2010 (Shaded areas are 95% CIs)

In Fig. 4, we present results from IE models for Japan and South Korea. These results indicate that the age groups most vulnerable to suicide differ across countries. In Japan, the effects of age were strongest in the middle-aged population, particularly among males in their fifties (β_*male*_ = 0.69, std. error = 0.036). For both males and females in Japan, suicide mortality increased steadily until age 50–54 before declining among males and leveling off among females. Conversely, in South Korea age had little effect on suicide mortality between ages 20 and 59 and the patterning of age coefficients was flat for both men and women. After age 60, the importance of age increased steadily for both men and women, peaking at the oldest age group (75–79) included in our analysis (β_*total*_ = 0.82, std. error = 0.075).

After a brief period of decline in suicide mortality between 1985 and 1990, period effects increased over the next decade in both countries (Fig. 4). In Japan, this increase was limited to the 1990s; period effects stabilized at an elevated level after 2000. The estimated period effects were similar among Japanese men and women, though the range in variation was much smaller among women. Unlike Japan, period effects in South Korea continued to increase in the new millennium, with rapid growth between 2000 and 2005 and an even stronger effect in 2010 (β_*total*_ = 0.77, std. error = 0.029). Both the magnitude and patterning of period effects was similar for men and women in South Korea.

Estimated cohort effects on suicide in Japan and South Korea reveal contrasting birth cohort patterns in these countries’ populations (Fig. 4). In Japan, the strongest effects were observed for the 1905–09 birth cohort (β_*total*_ = 1.00, std. error = 0.102), the eldest birth cohort in our analysis. These effects steadily weakened but remained significantly elevated for Japanese cohorts born between 1910 and 1924, before stabilizing near zero for several birth cohorts thereafter. Subsequently, cohorts born between 1945 and 1984 exhibited significantly lower rates of suicide mortality than other cohorts in Japan. This array of effects contrasts sharply with South Korea, where cohort effects were highest between the 1930s and the 1960s—corresponding roughly to the population born between Great Depression and the aftermath of the Korean War. Also different from the cohort patterns in Japan, the most recent birth cohorts in South Korea showed substantially lower rates of suicide mortality. Within each country, men and women exhibited similar patterns of cohort effects, though these effects were somewhat muted among women in South Korea.

## Discussion

Our study indicates that suicide mortality risks vary substantially across all three temporal dimensions (age, period, and birth cohort) in Japan and South Korea. According to Pampel [24], variation in suicide rates across demographic groups is an indicator of relative social and economic wellbeing, and related work has highlighted the continued importance of social integration during times of rapid change [5]. For instance, elevated odds of suicide mortality among middle-aged Japanese males suggest that this group might be subject to high levels of social stress, perhaps related to career demands at this stage of the life course. Similarly, high levels of suicide among Japanese cohorts born prior to 1920 imply that this group experienced significant social and/or economic strain, perhaps related to hardships induced by the Great Depression in early life, followed by World War II and reconstruction during this cohort’s midlife.

While elevated odds of suicide among certain demographic groups raise important public health concerns, they do not necessarily indicate that these groups have contributed materially to *changes* in suicide mortality over our period of observation. For example, over the past 20 years the percentage of Japan’s population between the ages of 40–65 has been remarkably stable (34.2 % in 1990; 34.4 % in 2000; and 34.0 % in 2010). Because this high-risk group has not increased in relative size between 1990 and 2010, the strong age effects associated with this group are not responsible for overall increases in Japan’s suicide rate. Also, despite alarmingly high suicide rates among Japanese cohorts born prior to 1920, these cohorts did not contribute to increasing general suicide rates because they comprise a rapidly diminishing fraction of Japan’s total population.

Conversely, while period effects in Japan were small in magnitude compared to the age and cohort effects, they nevertheless contributed to the general rise in suicide mortality because they were equally distributed across the entire Japanese population. Moreover, because period effects stabilized at a relatively high level between 2000 and 2010, overall rates of suicide mortality also stabilized at an elevated level and were not off-set by either age or cohort effects that have impacted certain subgroups in the Japanese population. Some studies have argued that increasing rates of suicide mortality in Japan were triggered by the economic crisis of the late 1990s [8]. Our results are clearly consistent with this argument and contrary to some previous claims that strong age or cohort effects are responsible for recent increases in suicide mortality in Japan [9].

Whereas modest period effects are primarily responsible for the 20 % increase in rates of suicide mortality in Japan between 1985 and 2010, multiple factors are responsible for the much larger 280 % increase in suicide mortality in South Korea. Age effects were particularly strong in the over-60 age group, which has comprised a fast-growing share of the total population in recent decades—from 6.8 % in 1985 to 15.9 % in 2010. Also, cohort effects in South Korea were strongest among persons born between 1920 and 1960, and these birth cohorts also make up a sizable fraction of South Korea’s total population. Finally—and perhaps most importantly—period effects on South Korea’s suicide rates have increased sharply and steadily since 1990. This means that suicide risks for the entire population of South Korea have increased markedly in the past 20 years. Thus, unlike trends in Japan’s suicide rates, which largely reflected period effects, the trends in South Korea’s suicide rates reflect the combined effects of age, birth cohort, and shared period effects in the population.

Previous research has emphasized high suicide rates among the elderly as well as cohort effects as the main reasons for the steep rise in suicide mortality rates in South Korea over the past 20 years [25]. In some regards our study supports those conclusions. However, our findings also point to recent secular changes—perhaps associated with rapid industrialization and population growth—in South Korea as a major contributor to increasing rates of suicide mortality.

### Policy implications

High rates of suicide have led to the development of suicide prevention programs in both Japan and South Korea. For example, in 2001 the Ministry of Health, Welfare and Labour in Japan launched a comprehensive suicide prevention campaign [26]. In line with this project, the Japanese government enacted the Community-based Prefectural Emergency Fund for Suicide Prevention in June 2009. Meanwhile, in South Korea, a national campaign for suicide prevention was launched in 2001 to improve public awareness of suicide and identify individuals who exhibit suicidal ideation. Recently, the South Korean government also established the Korean Association for Suicide Prevention and took measures to enforce the anti-suicide law in 2012.

Although we lack clear evidence on the efficacy of these suicide prevention programs, persistently high suicide mortality rates in Japan and South Korea indicate that more effective action is required. This is particularly true in South Korea where suicide rates appear poised to increase even further in the future without urgent public health action. One way to improve the effectiveness of public health efforts may be to direct limited public health resources towards the highest risk populations identified in our study. For example, while our study supports the call for renewed policy attention toward middle-aged males in Japan [9], it also shows that suicide risks are significantly elevated for women in this age range. Similarly, interventions that are specifically tailored for elderly individuals in South Korea could help ameliorate the very high rates of suicide mortality observed in this subpopulation.

In South Korea, it is paramount to identify the secular changes that have led to drastic increases in suicide mortality over the past two decades. Prior studies have investigated the influence of specific secular changes such as economic recession [8], unemployment [8], and divorce rates [6]. However, because any one of these factors is unlikely to fully explain the pattern of period effects in South Korea it may prove fruitful to investigate the combined effects of—and potential interactions between—various secular changes. Also, in South Korea serious challenges are posed by high suicide rates among cohorts born during the tumultuous era spanning the Great Depression and the Korean War. As members of these cohorts age, they may be subject to a form of “double jeopardy” as cohort-based experiences and age-related processes combine to increase the risk of suicide. The efficacy of policies aimed at the elderly in South Korea may improve by accounting for the difficult experiences shared by members of these birth cohorts.

### Strengths, limitations and future directions

To our knowledge, our study is the first to compare and contrast temporal dimensions of suicide in Japan and South Korea by applying the same statistical methods to data covering identical periods of observation. This approach led to the discovery that age, period, and cohort effects have made distinct contributions to changing rates of suicide mortality in these countries, despite their geographic proximity and certain cultural similarities. Differences in the demographic factors that have shaped suicide mortality in Japan and South Korea suggest that each country will benefit from locally applicable suicide prevention programs and also through additional research that can shed further light on the heterogeneity of factors affecting suicide in each country. Importantly, our study also extends previous APC research by including nearly a decade of data previously unexamined in South Korea. Given rapid increases in suicide rates in South Korea in recent years, our findings provide a much needed update on this issue and should provoke concern and further action among researchers and policymakers alike.

Despite these strengths, our study is limited by its inability to pinpoint precisely which social and economic factors are responsible for the age, period, and cohort effects that we observe. Given the data available to address the specific research objectives in this study (i.e., vital statistics and census data), we did not have access to socioeconomic or other potentially useful information (e.g., marital status, urban/rural residence, or familial and social integration) that could further improve our understanding of suicide mortality in Japan and South Korea. Future studies should build upon the results of our investigation by attempting to identify these factors. For example, contingent on the availability of individual-level suicide data (e.g., in a prospective cohort study where mortality is carefully monitored over a long period of observation), researchers could explore hierarchical APC models, which would facilitate the simultaneous examination of both macro-level factors (e.g., unemployment rates) that may underlie period or cohort effects, as well as micro-level factors (e.g., depression) that are more proximate determinants of suicide [5]. Whichever research strategies are adopted, it is essential that future research continue to investigate the determinants of suicide in Japan and South Korea. This should be an urgent national priority in each country, as a deeper understanding of these issues will lead more effective policies, ultimately reducing the burden of suicide and improving population health for all.

## Conclusion

In spite of cultural, demographic and geographic similarities in Japan and South Korea, the underlying causes of increased suicide mortality rates in these two countries differ. In Japan, the increase in suicide mortality is primarily driven by modest period effects, initiated during the Asian financial crisis of the late 1990s. In South Korea, the temporal factors that underlie the stark increase in suicide mortality are more complex, including recent secular changes, pronounced age effects among the elderly individuals, and strong cohort effects for those born between the Great Depression and the aftermath of the Korean War. Our findings suggest that public health responses should be tailored to fit each country’s unique situation.

### Ethics

The institutional review board at Utah State University (USU) determined that this project does not qualify as human subjects research and is exempt from institutional oversight (USU Assurance: FWA#: 00003308, Protocol#: 4071).

### Consent to publish

Not applicable.

### Availability of data and materials

Data provided by Statistics Korea are publicly available at http://kosis.kr/statHtml/statHtml.do?orgId=101&tblId=DT_1B34E01&conn_path=I2&language=en. Data provided by Vital, Health and Social Statistics Division of Ministry of Health, Labour and Welfare in Japan are publicly available at http://www.stat.go.jp/english/data/chouki/02.htm.

## Abbreviations

APC: age-period-cohort
CGLIM: constrained generalized linear model
IE: intrinsic estimator
OECD: organization for economic cooperation and development
PYL: person-years lived

## Footnotes

1 The second identification issue potentially applicable to tabular data arranged in an age-by-period matrix is that A, P and C are linearly related to the outcome variable, Y. However, because equation (1) specifies A, P and C as categorical rather than continuous variables, no assumptions are made regarding the functional form of associations between Y and these temporal dimensions [19]. In such a model, the second identification issue only holds when the categorical variables reveal *exactly* linear associations between A, P, C and Y. In practice, it is exceedingly rare to observe such perfectly arranged associations— and it is clearly not applicable in any of the APC models in our investigation.

2 Because this process is only affected by *a* and *p*, some scholars have endorsed it as a non-arbitrary way to constrain the model and identify a unique set of APC estimates [20]. Moreover, while IE and CGLIM yield similar point estimates when *a priori* assumptions are valid, IE provides superior statistical efficiency [12].

3 According to Held and Riebler [27], rotation among the APC coefficients can occur without altering model fit by using different referent categories in IE models. The *apc*_*ie* module in Stata uses the last APC categories as reference groups by default; to check the extent of rotation, we estimated four additional models using *ie*_*rate*/*ie*_*norm* modules [28]. Each model used different A-P-C groups as referent categories. For one such model, we used the first groups (1-1-1), and for the other three models we used randomly selected groups (14-4-14, 9-4-3, 11-4-2) of A-P-C. Our results show negligible coefficient rotation, meaning that the patterning of APC effects was nearly identical across all five models.
